# RETRACTED: Frolli et al. Children on the Autism Spectrum and the Use of Virtual Reality for Supporting Social Skills. *Children* 2022, *9*, 181

**DOI:** 10.3390/children13081038

**Published:** 2026-08-04

**Authors:** Alessandro Frolli, Giulia Savarese, Francesca Di Carmine, Antonia Bosco, Emilio Saviano, Angelo Rega, Marco Carotenuto, Maria Carla Ricci

**Affiliations:** 1 Disability Research Centre, University of International Studies in Rome, Via Cristoforo Colombo, 200, 00147 Rome, Italy; 2 Department of Medicine and Surgery, University of Salerno, 84084 Salerno, Italy; 3 FINDS—Italian Neuroscience and Developmental Disorders Foundation, 81040 Caserta, Italy; 4 Department of Humanities, University of Naples, 80133 Naples, Italy; 5 Clinic of Child and Adolescent Neuropsychiatry, Department of Mental Health, Physical and Preventive Medicine, Università degli Studi della Campania “Luigi Vanvitelli”, 80100 Naples, Italy

The journal retracts the article titled “*Children on the Autism Spectrum and the Use of Virtual Reality for Supporting Social Skills*” [1], cited above.

Following publication, concerns were raised with the Editorial Office regarding the data sample and the ethical approval information reported in this publication [1].

In accordance with the journal’s standard procedures, the Editorial Office and Editorial Board conducted an investigation during which questions were raised about the data sample and the lack of appropriate Institutional Review Board approval, prior to participant enrolment. The authors cooperated with the investigation and provided additional information relating to the data sample and the ethical approval. However, after careful consideration, the Editorial Board determined that the explanations and supporting documentation did not sufficiently address the remaining concerns about the ethical approval information and that the study is non-compliant with MDPI’s policies for Research Involving Human Subjects. As a result, the Editorial Board has decided to retract this paper as per MDPI’s retraction policy.

This retraction was approved by the Editor-in Chief of *Children*.

The authors have been informed of this retraction and have indicated their disagreement with this decision.
